# The effect of a 5-week therapeutic massage on erector spinae and upper trapezius muscle stiffness as determined by shear-wave elastography: a randomized controlled trial

**DOI:** 10.3389/fspor.2024.1428301

**Published:** 2024-08-26

**Authors:** Amadej Jelen, Erina Javornik, Sara Gloria Meh, Žiga Kozinc

**Affiliations:** Faculty of Health Sciences, University of Primorska, Izola, Slovenia

**Keywords:** massage, muscle stiffness, elastography, upper trapezius, erector spinae

## Abstract

**Introduction:**

Massage is an effective treatment for reducing pain, swelling, stiffness, and improving muscle mobility. Although self-reported benefits on muscle stiffness and pain are well-known, studies measuring muscle stiffness objectively are scarce.

**Methods:**

A randomized controlled trial involving 30 recreationally active young women (22.3 ± 0.4 years) was conducted. The participants were randomly assigned to either the control group or the intervention group which received a series of five 30-min whole back therapeutic massage sessions over 5 weeks. Shear wave elastography was used to assess muscle stiffness (erector spinae (ESp) and upper trapezius (UT) muscles) before and after the intervention and at 3-week follow-up.

**Results:**

For ESp, there was no statistically significant time × group interaction (*F* = 2.908; *p* = 0.063). However, there was a statistically significant and large time × group interaction for UT (*F* = 13.533; *p* = 0.006; *η*^2^ = 0.19). *Post-hoc* testing for time indicated that the shear modulus in the intervention group was reduced at follow-up (*p* = 0.005; *d* = 1.02), while the difference between baseline and post-intervention measurements were not statistically significant (*p* = 0.053; *d* = 0.75).

**Conclusion:**

In conclusion, massage significantly reduced proximal UT stiffness both 3 days and 3 weeks after the intervention. However, it had no significant effect on the distal part of UT or the ESp muscle.

## 1 Introduction

Massage therapy, recognized for its historical significance and established benefits, is acknowledged for its capacity to alleviate pain, diminish swelling, lessen stiffness, and enhance muscle mobility (1, 2). The fundamental objective of therapeutic massage is to target and resolve distinct physical concerns, such as chronic pain, injuries, or muscle imbalances (3). This may entail a focused and intense approach, concentrating on specific areas of concern to alleviate discomfort (4). Therapeutic massage incorporates many techniques, including surface effleurage (light friction technique), deep effleurage, deep rubbing, petrissage (kneading, wringing, skin rolling), deep petrissage, and parts of trigger point therapy (5, 6).

The ability of massage to reduce muscle stiffness could be clinically significant because muscle stiffness can contribute to the development or ongoing presence of pain (7). Rafsanjani Deh Qazi et al. (8) reported in their systematic review that lumbopelvic muscle stiffness was significantly higher in patients with lower back pain than in those with no pain in the lower back. There has been also an established relationship between lumbar multifidus muscle stiffness and lower back pain (9). Furthermore, higher neck muscle stiffness has been associated with neck pain in migraine patients (10), patients with chronic neck pain (11), office workers (12), and also female employees in general (13). Additionally, two recent meta-analysis reported increased stiffness in back and neck muscles (14, 15). While this is suggesting that stiffness relief could potentially contribute to resolution of pain and decrease the chances of experiencing similar issues in the future, it has to be emphasized that all evidence on relationship between pain and stiffness is cross-sectional. While a positive effect of massage on self-reported stiffness and pain is well-documented, there is a lack of studies assessing its effect on objectively measured muscle stiffness (16).

Shear wave elastography is an emerging method for assessing the elasticity of soft tissues based on acoustic radiofrequency force impulse (17). Briefly, the impulse generates transversely-oriented shear waves that propagate through surrounding tissue (18). Stiffer tissues represent higher velocities of shear waves, from which shear modulus (measured in kilopascals, kPa) is determined by assuming a consistent tissue density, with a standardized value of approximately 1,000 k/m^3^ applied specifically to muscle tissue (19, 20). A recent scoping review of Cipriano et al. (21) has reported that shear-wave elastography is being extensively used in research on variety of traumatic and pathological conditions affecting soft tissues in the musculoskeletal system including muscle (22), tendons (23), ligaments (24) and nerves (25). However, the application of this method in discerning the impact of massage on muscle stiffness has been limited. Given that measuring muscle stiffness with shear wave elastography provides the most objective and accurate assessment currently available, it is likely to become more prevalent in future studies and applications.

Studies exploring the effects of massage on musculotendinous stiffness show inconsistent findings. These inconsistencies may be linked to variations in the muscles studied, the type of massage administered, and differences in the duration and intensity of massage. For instance, no significant effects on stiffness were found with a brief foam rolling session on the pectoralis muscle (26), 1-min percussive massage on Achilles tendon (27), 5-min alternating classical massage on masseter and temporalis muscles (28), as well as 5-min classical and sports massages on each erector spinae (ESp) and upper trapezius (UT) muscle (29). The impact observed in certain studies could potentially have been significant with the inclusion of a larger participant group. For example, the grou*p* × time interaction was approaching statistical significance (*p* = 0.073) in a study involving a modest sample size (*n* = 15) (29). In contrast to the aforementioned studies, a statistically significant reduction in the stiffness of the medial gastrocnemius was observed following a 5-min classical massage (30), 7-min classical massage (31) and a 7-min warm-up sports massage (32). Seven-minute classical massage also decreased muscle stiffness of the biceps brachii (31).

Thus far, most of the research indicates that the reduction in muscle stiffness tends to be temporary, typically lasting only a few minutes. For example, observations have highlighted that the stiffness in the medial gastrocnemius were resorted to baseline within 3–5 min following a classical massage (30, 31), 5 min after percussive massage in the Achilles tendon (27). Also, effects of 7 min of classical massage on biceps brachii wore off in less than 5 min after the massage. Conversely, findings from another study indicated a potential for a more enduring effect, revealing the lowest muscle stiffness recorded 10 min after both classical and sports massage (29). This implies that the influence of massage on muscle stiffness, particularly in the ESp and UT areas, may extend for at least 10 min. This provides a promising prospect for alleviating discomfort in individuals experiencing chronic pain in the back and neck regions. To the best of our knowledge, only one study has examined long-term impacts of massage, reporting that a 3-min hamstring musculotendinous junction self-massage (5 times a week for 12 weeks) did not have an effect on hamstring stiffness (33). However, no study has been conducted to explore the long-term effects of massage on back or neck muscle stiffness. Therefore, the aim of this study was to investigate the chronic effects of therapeutic massage on ESp and UT muscle stiffness, measured by shear-wave elastography on young recreationally active women. The null hypothesis for the study was that there will be no statistically significant difference in muscle stiffness changes over time between therapeutic massage and control groups. The alternative hypothesis was that there would be a statistically significant reduction in muscle stiffness in the massage group compared to the control group after a 5-week intervention period, with a statistically significant time × group interaction). In other words, we hypothesized that the therapeutic massage group would exhibit a significant decrease in stiffness of the ESp and UT muscles, while the control group would show no significant change.

## 2 Methods

### 2.1 Sample size calculation

Given the lack of long-term studies investigating the effects of massage on muscle stiffness, we determined the estimated required sample size using the effect sizes from studies investigating acute effects. In a previous study, Cohen's d values 10-min after massage application for ES and UT were 0.63–0.85 (29). Assuming a null effect in the control group in our study, the lowest Cohen's *d* value (0.63) was converted to Cohen's f measure, which is a suitable for sample size calculation for interaction effects (*f* = 0.31). We used G*Power 3.1 software (Heinrich Heine University, Düsseldorf, Germany) for calculating sample size for the interaction effect of within-between analysis of variance (ANOVA) [effect size (f) = 0.31; *α* error = 0.05, and power = 0.80; correlation between repeated measures = 0.5]. The sample size calculation indicated that at least 12 participants per group were needed for the study. To increase the power of the study and account for potential drop-outs, a total of 30 participants were enrolled for baseline assessment.

### 2.2 Participants

We included a sample comprising 30 healthy and physically active women who volunteered for the study, with an average age of 22.3 years (±0.4), a mean height of 166.6 cm (±1.2), and an average body mass of 61.4 kg (±1.5). Participants were recruited through invitations on Faculty's website and social media. No financial or other incentives were offered to the participants. Participants were eligible to participate if they (a) had no history of current or prior neck or back injuries, musculoskeletal issues, myopathies, or neurological disorders (b) reported to engage regularly in physical activity (at least 150 min per week). Prior to their involvement, the participants received comprehensive information about the research objectives and procedures and provided voluntary consent through a signed declaration. Additionally, participants were instructed to avoid intense resistance exercise for a minimum of 72 h and any exercise for a minimum 24 h before each measurement session. The study's non-invasive research methods and interventions were ethically reviewed and approved by the University of Primorska's Commission for Ethics in Human Subjects Research (approval number: 4264-19-6/23).

### 2.3 Study design and procedures

To ensure balanced group sizes, our study employed a block randomization strategy with a predetermined block size (15 per group), aiming for an equal distribution of participants across the treatment and control groups. This approach was chosen to maintain statistical power and minimize selection bias. Following baseline measurements, participants were randomly assigned to their respective groups through a blind draw from sealed envelopes. The group allocation, concealed within these envelopes, was disclosed only to the examiner responsible for administering the massage. To minimize the potential impact of circadian rhythms, each participant attended all measurements at roughly the same time each day (±1 h). All evaluations took place in a stable setting—an air-conditioned room with temperatures controlled within the range of 22 °C–23 °C.

Participants were invited to the laboratory on three separate occasions for the assessment of muscle stiffness. Following the initial measurement, a 5-week intervention period was initiated. During this period, the massage group underwent five sessions of therapeutic massage, with attempts to space these sessions as evenly as possible. The second measurement was conducted 3 days after the final massage session for the intervention group, or equivalently, 3 days after the conclusion of the intervention period for the control group. The third and final measurement took place 3 weeks after the last massage session for the intervention group, or 3 weeks after the intervention period ended for the control group.

On the assessment visits, the participants were instructed to lie face down on the massage table and completely relax for a 10-min period before the measurements commenced. This step was taken to minimize the impact of prior activities on muscle stiffness. Subsequently, shear-wave elastography was used to assess the stiffness of erector spinae (superior and inferior point) and upper trapezius (proximal and distal point) on both sides of the body. The order of the assessed points remained consistent across all sessions.

### 2.4 Intervention

The massage intervention was administered by a skilled kinesiologist holding a graduate degree, possessing extensive experience in applying diverse massage techniques. The massage lasted 30 min including 18 techniques each lasting 1 min, encompassing the entire back region. Six techniques were done on both sides simultaneously and 12 on one side at a time. Pressure applied during massage was categorized on a 5-point scale, from light lotioning (grade I), heavy lotioning (grade II), medium pressure (grade III), strong pressure (grade IV) and deep pressure (grade 5). Similarly, we defined massage tempo from extremely slow pace (grade I), slow pace (grade II), moderate pace (grade III), fast pace (grade IV), and rapid pace (grade V) (29). We employed this system consistently to delineate the level of pressure and the pace for each massage technique throughout the study.

Used techniques were: surface effleurage (I, IV) with the whole palm around the back, kneading (IV, II) with the root of the palm in the region of the lower back in the direction of the spine, deep effleurage (III, III) with the little finger side of the palm in the direction from the head to the feet, deep effleurage (III, III) with the tip of the fingers in the area of trapezius muscle, deep effleurage (III, III) with the thumb side of the palm from the direction of the acromion towards the C7 vertebra, deep rubbing (III, III) with the knuckles away from the spine from legs to the head and vice versa, rubbing (II, III) with little finger side of the fist parallelly to the spine on ESp throughout the entire muscle length, deep petrissage (IV, II) with the root of the palm at an angle of 45° to the spine in the region of lower back, deep rubbing (III, III) with knuckles around scapula on trapezius region with examinee's hand behind the back, deep effleurage (III, III) with the thumb side of the palm from the direction of the acromion towards the C7 vertebra, deep rubbing (IV, II) with the knuckles from the direction of the C7 vertebra towards the acromion on both sides simultaneously, surface effleurage (II, II) with thumbs on neck part of UT muscle on both sides, deep effleurage (II, III) with whole palm on ES throughout the entire muscle length in direction from head to legs on both sides, deep petrissage (III, III) with finger tips on quadratus lumborum and lower back part of ESp, kneading (IV, II) the whole UT perpendicular to its course with the fingers, deep rubbing (IV, III) with the little finger side of the palm and the knuckles of the other hand in the direction from the head to the feet throughout the entire ESp muscle length, deep effleurage (III, III) with thumb side of the palm from the direction of the C7 vertebra towards the acromion on both sides simultaneously and deep effleurage (II, IV) with the whole palm around the back.

### 2.5 Muscle stiffness assessment

**Figure 1:**
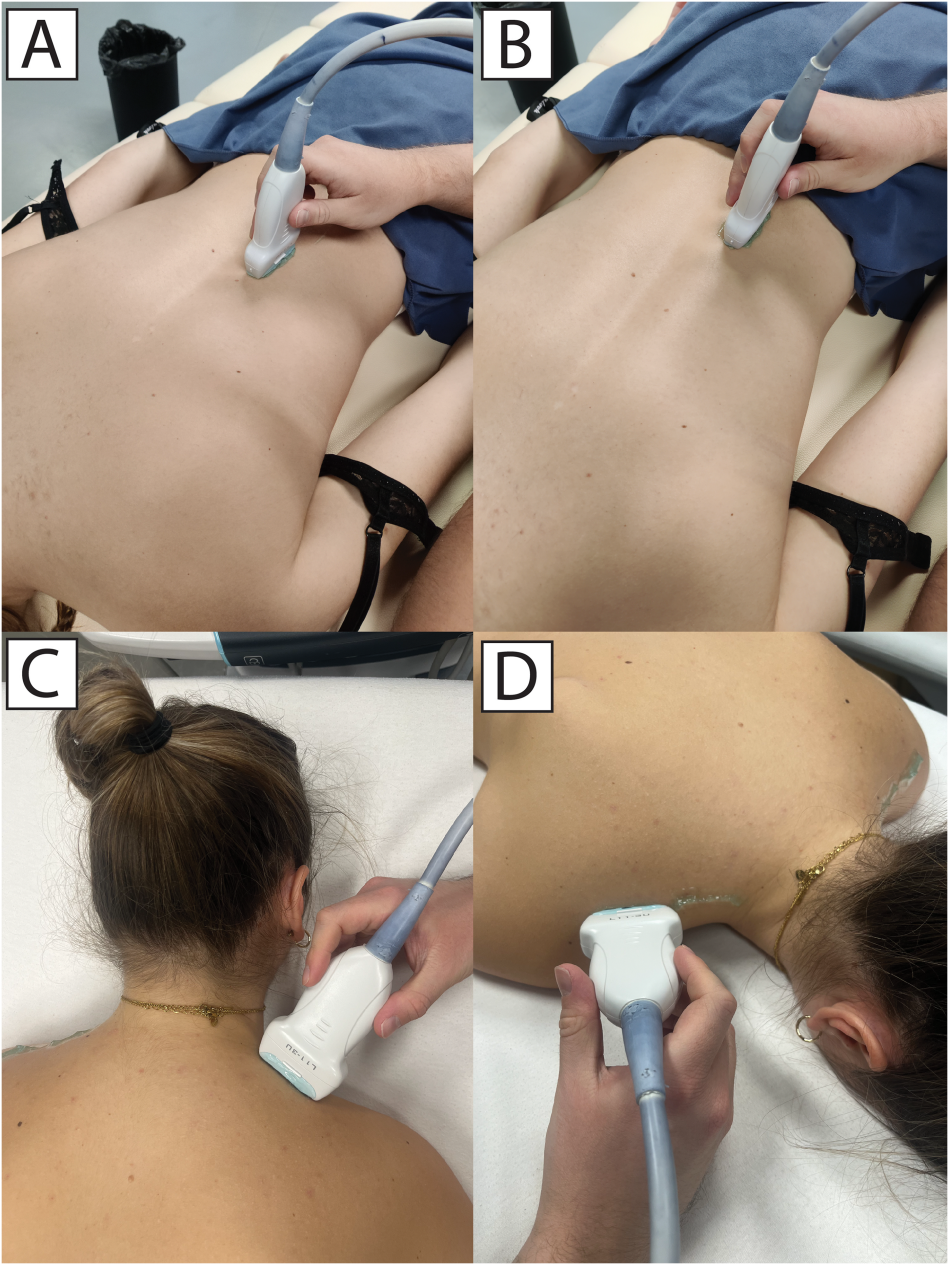
Measurements of erector spinae at superior **(A)** and inferior **(B)** location, and upper trapezius at proximal **(C)** and distal **(D)** locations.

**Figure 2:**
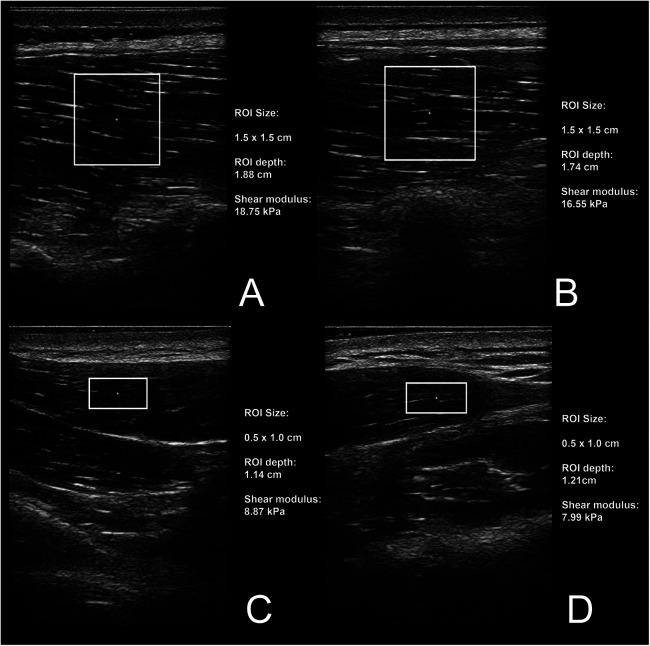
Snapshots of ultrasound measurements for superior **(A)** and inferior **(B)** location on erector spinae muscle, and proximal **(C)** and distal **(D)** point on upper trapezius muscle.

Muscle stiffness assessment was carried out by a skilled examiner proficient in shear-wave elastography, who remained unaware of the randomized conditions. Muscle stiffness was assessed through shear wave elastography using the Resona 7 diagnostic ultrasound system from Midray, located in Shenzhen, China. The system was configured to Musculoskeletal Mode, assuming a muscle tissue density of 1,000 kg/m^3^ (34). A medium-sized linear probe (model L11-3U, Midray, Shenzhen, China) was employed, along with a water-soluble hypoallergenic ultrasound gel (AquaUltra Basic—Ultragel, Budapest, Hungary). The designated area for analysis was established at 15 × 15 mm for ESp and 5 × 10 mm for UT. To prevent the inclusion of non-muscular tissue, the depth of the region of interest was customized for each subject and documented for future assessments. The measurement locations were consistently maintained across all visits for each participant. For both the ESp and UT, we assessed two distinct locations on each muscle. In the case of the ESp, the probe was positioned along the Th10 (superior position) and Th12 (inferior position) vertebrae line, focusing on the medio-lateral point of greatest prominence (Figures 1A,B). In the case of the UT, the probe was aligned parallel to the muscle fibers and the first location was 1 mm lateral to the neck (proximal), and the second location was 3 cm laterally to the first, ensuring we targeted the muscle's distal section (distal) (Figures 1C,D). Minimal pressure was applied to the skin using the probe, maintaining constant contact between the ultrasound gel, skin, and probe. Muscle stiffness was quantified using shear modulus, measured in kilopascals (kPa). Following data collection, entries were promptly recorded in prearranged tables. Two sets of measurements were conducted for each trial, each comprising eight consecutive scans. The resulting mean value was computed for each set. Further analysis involved taking the mean value derived from the two measurement sets. The software instantly presented shear modulus values, which were then transcribed for subsequent use. Snapshots of ultrasound scans are shown in Figure 2.

### 2.6 Statistical analysis

The data are presented as means ± standard deviations. The normality of the data distributions for all variables was verified with Shapiro-Wilk test (all *p* ≥ 0.068) and visual inspection of histograms and Q-Q plots. Intra-class correlation coefficients (ICC; single measures, absolute agreement) were calculated to assess reliability of shear modulus measurements at each time points (i.e., the reliability was calculated the between two trials at each time point). Given the small sample size, we also considered 95% confidence intervals (CI). An ICC values lower than 0.50 were considered as indicative of poor reliability, values between 0.50 and 0.75 as moderate reliability, values between 0.75 and 0.90 as good reliability, and values greater than 0.90 as excellent reliability (35). For each muscle, we used general linear model with side (left, right), location (inferior and superior for ESp; proximal and distal for UT), and time (baseline, post-intervention and follow-up) as within-participant factors and group (intervention, control) as a fixed between-participant factor. The primary interest was in the interaction between group and time effects, indicating a potentially different response of the groups through time; therefore, estimated marginal means of the group × time interaction with 95% CI were considered as the primary statistical outcome. These values demonstrate the difference between groups in time, pooled across sides and location. The *η*^2^ values were considered as measures of effect sizes, indicating no effect (<0.01), a small effect (0.01–0.039), a medium effect (0.06–0.14) and a large effect (>0.14). In case of significant interactions, Bonferroni-corrected *post-hoc* testing with univariate analysis of variance and *t*-tests were done, with Cohen's *d* calculated to assess effect sizes for the latter. Cohen's *d* were considered as trivial (<0.20), small (0.20–0.50), medium (0.50–0.80) and large (>0.80) (36). The threshold for statistical significance was set at *α* < 0.05 and all analyses were carried out in SPSS statistical software (version 25.0, IBM, USA).

## 3 Results

**Figure 3:**
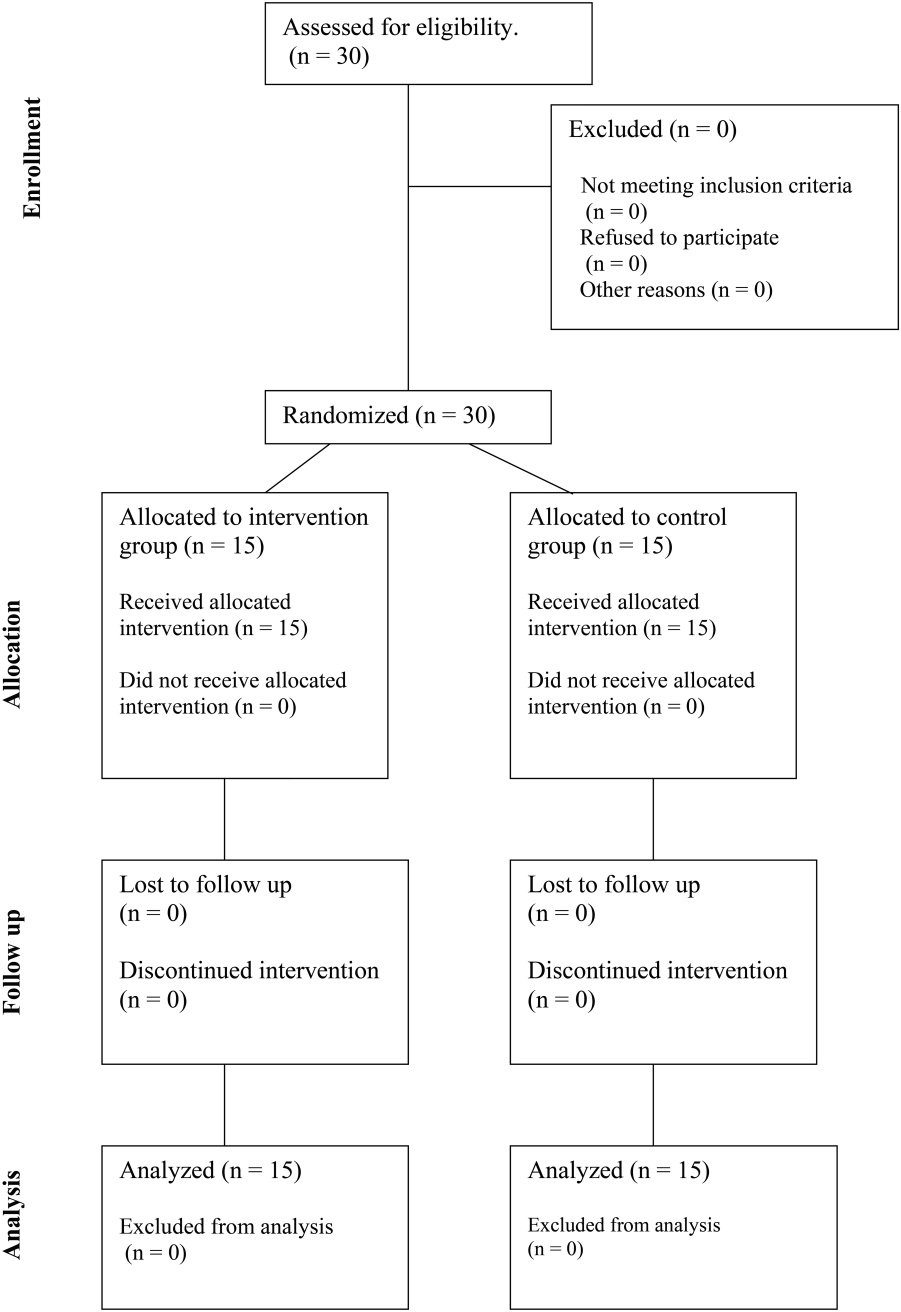
CONSORT diagram showing the flow of participants through each stage of a randomized trial.

The CONSORT flowchart of the participants is shown in Figure 3. There were no instances of participant dropout at any stage of the research process, and all participants received the allocated massages sessions according to schedule. Furthermore, no troubles or side-effects were observed or reported by the participants in relation to the study's procedures or interventions.

### 3.1 Reliability

The reliability of the shear modulus measurements is shown in Supplementary File 1, separately for each muscle, location, side and timepoint in the study (24 variables in total). ICC point estimates indicated either good (ICC = 0.77–0.89; 9 variables) or excellent (ICC = 0.90–0.99; 15 variables) reliability. Considering the 95% CI, 19/24 variables exhibited good to excellent reliability (lower bound CI: 0.76–0.97; upper bound CI: 0.94–0.99), 4/24 variables exhibited moderate to excellent reliability (lower bound CI: 0.63–0.74; upper bound CI: 0.90–0.94), and one variable had moderate to good reliability (lower bound CI: 0.57; upper bound CI: 0.88).

### 3.2 Effects of massage on ES shear modulus

For the ESp muscle, there were no statistically significant main effects of location (*F* = 0.780; *p* = 0.385), side (*F* = 3.990; *p* = 0.055), time (*F* = 2.128; *p* = 0.129) and group (*F* = 3.159; *p* = 0.086). There were no 4-way or 3-way interactions (all *p* < 0.135), and there was also no statistically significant time × group interaction (*F* = 2.908; *p* = 0.063), indicating that the responses of the two groups did not statistically differ as a function of time. There was a significant side × location interaction (*F* = 6.548; *p* = 0.016; *η*^2^ = 0.19), indicating that side-to-side differences depend on location of the measurement; however, given that this was not the primary interest of the study, further analyses were not carried out.

### 3.3 Effects of massage on UT shear modulus

For the UT shear modulus, there was no statistically main effect of side (*F* = 1.441; *p* = 0.240), and there were no 4-way or 3-way interactions (all *p* < 0.296). There was a statistically significant and large effect of location (*F* = 18.832; *p* < 0.001; *η*^2^ = 0.40), with proximal part of the UT displaying lower values compared to distal UT (marginal mean: 6.60 kPa (95% CI: 6.15–7.04 kPa) for proximal part; 7.55 kPa (95%CI: 7.04–8.07 kPa) for distal part). The main effects of group (*F* = 1.211; *p* = 0.281) and time (*F* = 3.107; *p* = 0.053) were not statistically significant; however, there was a statistically significant and large time × group interaction (*F* = 13.533; *p* = 0.006; *η*^2^ = 0.19), indicating that the values in each group changed differently in time. In addition, there was a statistically significant and large locatio*n* × time interaction (*F* = 5.583; *p* = 0.011; *η*^2^ = 0.17), indicating that the changes in time were dependent on the measurement location. The estimated marginal means for time × group interaction with 95% CIs are shown in Table 1. *Post-hoc* testing for time indicated that the shear modulus in intervention group was reduced at follow up (*p* = 0.005; *d* = 1.02), while the difference between baseline and post-intervention measurements were not statistically significant (*p* = 0.053; *d* = 0.75). The difference between post-intervention and follow-up was also not statistically significant (*p* = 1.000). In the control group, there were no pair-wise statistically significant differences among time points (*p* = 0.391–1.000).

**Table 1:** Estimated marginal means for UT shear modulus—interaction between group and time.

Group	Time	Mean	SD	95% CI	
				Lower bound	Upper bound
Intervention	Baseline	7.47	2.10	6.72	8.21
	Post-intervention	6.57	1.99	5.85	7.28
	Follow -up	6.52	1.69	5.92	7.13
Control	Baseline	7.19	2.03	6.44	7.94
	Post-intervention	7.61	1.98	6.89	8.32
	Follow -up	7.13	1.91	6.53	7.74

SD, standard deviation; CI, confidence interval.

Given the interaction between location and time, we also explored the estimated marginal means for locations across time, which is shown in Table 2. The values indicate that the statistically significant reduction in stiffness occurred in the proximal part of the UT. *Post-hoc* testing within intervention group indicated a statistically significant reduction of shear modulus in proximal UT during post-intervention (*p* = 0.038; *d* = 0.73) and follow-up (*p* = 0.003; *d* = 1.07) compared to baseline, while there was no difference between the post-intervention and follow-up (*p* = 1.000). However, no differences between time points were statistically significant for the distal UT (*p* = 0.603–0.791). In the control group, there were no differences across time points for either proximal UT (*p* = 0.309–1.000) or distal UT (*p* = 0.173–1.000).

**Table 2:** Estimated marginal means for UT shear modulus—interaction between location and time.

Group	Location	Time	Mean	SD	95% CI	
					Lower bound	Upper bound
Pooled	Proximal	Baseline	7.17	1.61	6.59	7.76
		Post-intervention	6.56	2.08	5.84	7.28
		Follow -up	6.06	1.11	5.67	6.45
	Distal	Baseline	7.47	1.73	6.85	8.10
		Post-intervention	7.60	1.57	7.06	8.15
		Follow -up	7.58	1.60	7.01	8.15
Intervention	Proximal	Baseline	7.33	1.37	6.57	8.08
		Post-intervention	5.95	1.55	5.09	6.80
		Follow -up	5.77	0.79	5.33	6.21
	Distal	Baseline	7.60	1.16	6.95	8.24
		Post-intervention	7.18	1.08	6.58	7.78
		Follow -up	7.26	1.24	6.57	7.95
Control	Proximal	Baseline	7.02	1.73	6.06	7.98
		Post-intervention	7.18	2.23	5.94	8.41
		Follow -up	6.35	1.24	5.67	7.04
	Distal	Baseline	7.35	2.06	6.21	8.50
		Post-intervention	8.03	1.75	7.06	9.00
		Follow -up	7.90	1.75	6.93	8.87

## 4 Discussion

This randomized controlled trial investigated the effects of 5 therapeutic massage sessions on the stiffness of the ESp and UT muscles. The reliability of shear modulus measurements was confirmed as good to excellent across most variables, ensuring the robustness of our findings. Our primary findings are twofold: First, massage did not significantly alter the stiffness of the ES muscle. Second, for the UT muscle, massage resulted in a significant reduction in stiffness, but only in the proximal part. This outcome likely reflects the tendency of massage techniques to focus more on the proximal rather than the distal areas of the UT muscle.

The findings of this study suggest that benefits of massage on objectively-measured muscle stiffness of proximal part of UT endure for a minimum of 3 weeks. Prior study indicated that the effects of classical massage on muscle stiffness of medial gastrocnemius reverted to baseline within a mere three to 15-min timeframe (30, 31), questioning the long-term utility of massage. To the best of our knowledge, there has been only one study that investigated the long-term effects of massage (33). This particular study focused on the prolonged effects of a 3-min self-administered massage targeting the musculotendinous junction of the hamstrings. The massage routine, carried out five times a week over a span of 12 weeks, did not have a significant impact on the stiffness of the hamstrings (33), which is in contrast to our findings. In addition to muscle-specific responses to massage, a potential reason for the lack of significant impact on hamstring stiffness could be attributed to the absence of professional massage administration (37, 38). The cumulative weekly massage time of 15 min, with each session lasting just 3 min, may be too brief for effective treatment, possibly limiting the benefits of more extended, thorough sessions. There is no documented evidence suggesting that massage sessions shorter than 5 min significantly reduce muscle stiffness (26–28). It could be that longer sessions, such as 30 min as used in this study, are needed to effectively decrease muscle stiffness. In a similar vein, literature suggests that lower volumes of static stretching only affect the stretch tolerance, while prolonged and intense stretching is needed to change muscle mechanical properties such as muscle stiffness (39).

A recent study reported that achieving a positive impact on the UT muscle stiffness may require the application of stronger pressure during massage sessions (29). Based on these findings, the level of pressure is the current study was purposefully set to be firmer than typical for a classical massage, but gentler than typically used for sports massage. Interestingly, the former study indicated that ESp muscle requires less pressure for positive effects (29). Therefore, it seems surprising that our study observed an effect on the UT muscle but not on the ESp muscle. However, there was nearly significant group × time interaction for ESp (*p* = 0.063). Exploratory *post-hoc* testing for time indicated that the shear modulus in intervention group was reduced not statistically significantly after the intervention (*p* = 0.072) or at follow-up (*p* = 0.135), despite moderate effect sizes (*d* = 0.72 and 0.69, respectively). In the control group, there effect sizes were negligible (*d* = 0.02 and 0.09; *p* = 1.000). This hints that the lack of findings for these muscles might be due to a small sample size rather than the massage intensity being inadequate, and further research is warranted.

The elapsed time since the last massage could be an important factor in detecting changes in muscle stiffness. In their study, Akazawa et al. (33) conducted post-intervention muscle stiffness assessments 24 h after the last massage. A majority of the studies examining other methods to reduce muscle stiffness failed to specify the duration that had passed since the intervention (26, 40). To minimize acute effects and focus on chronic impacts of massage on muscle stiffness, our study allowed a 72-h interval after the last massage session. Weekly frequency was set to 1 session, as this seemed to be realistic for practical application. Despite the low volume and frequency of massage, noteworthy effects were observed in terms of reducing muscle stiffness in UT, suggesting the intervention volume was sufficient. In contrast, studies on stretching (40, 41) and foam rolling (26, 42), reported mixed results for muscle stiffness reduction in various muscles, with no clear correlation between stretching duration and stiffness levels. While larger volumes of stretching are likely needed to elicit changes in muscle stiffness (39), the effects seem to be dependent on the nature of the intervention and the specific muscle under consideration (26, 40–43). Further studies should also explore the importance of weekly frequency and duration in massage interventions.

The prolonged effects observed 3 days post the final massage session could be attributed to several mechanisms that exert a sustained impact on muscle stiffness (44). Persistent muscle tension, chronic pain and aging has the potential to bring about alterations in the structure of muscle fibers over time (45). Massage could play a role in influencing the remodeling of muscle fibers (46). It has also the potential to alleviate mechanical hyperalgesia, indicating that with repeated sessions, the body may adjust to a decreased sensitivity to pain (47). This adaptation may lead to reduced muscle stiffness as the protective response to pain decreases. However, since our study involved pain-free participants, other mechanisms likely played a role. Moreover, engaging in 30-min massage sessions twice a week over a 5-week period has been observed to lead to a reduction in salivary cortisol levels (48). Additionally, there is some indication that massage therapy enhances parasympathetic activity (49) and reduce systemic inflammatory responses (50, 51). Myofascial treatment sessions have the capacity to improve microcirculation, enhancing the flow of blood at the capillary level, which results in better transportation of nutrients and oxygen to the muscles, and it facilitates the effective removal of waste products (52). These outcomes could potentially have long-term benefit in mitigating muscle stiffness. Future research should aim to explore the specific mechanisms through which massage influences muscle stiffness.

Certain limitations of this research should be acknowledged. Firstly, the awareness of participants about receiving massage introduces potential bias. However, the lack of blinding in massage treatments is expected to continue as a fundamental challenge in conducting research in this area. Furthermore, participants were instructed to avoid intense resistance exercise for a minimum of 72 h and any exercise for a minimum 24 h before each measurement session, but they could not avoid college classes, which might have increased ESp muscle stiffness due to prolonged sitting, potentially affecting the measurements (53). Another limitation of this study is the lack of data on participants’ daily physical activity over the course of the intervention, which could influence muscle stiffness. Although randomization should minimize differences between the groups, future studies should control for physical activity to better isolate the effects of therapeutic massage. Additionally, while we established a classification system for massage pressure and tempo and implemented supervision to maintain consistency, we did not use objective measures to validate the correct application of these parameters. Future research should incorporate objective validation techniques to ensure the accuracy and reliability of massage protocols. Lastly, our study comprised exclusively young, healthy, recreationally active females. These limitations narrow the applicability of our results to more extensive populations, including males, various age categories, and notably, individuals with specific health conditions. We assume that the impact of a massage tends to be more pronounced for individuals with greater muscle stiffness. However, further investigation is required to explore this assumption.

## 5 Conclusion

In conclusion, our findings indicate that a series of five 30-min therapeutic massage sessions performed over 5 weeks lead to a significant reduction in muscle stiffness, specifically within the proximal part of the UT. However, this observed decrease in stiffness was not replicated in the distal part of the UT or the ESp muscle. It is noteworthy to emphasize that the decrease in muscle stiffness observed in the proximal part of the upper trapezius endured for a minimum of 3 weeks following the conclusion of the last therapeutic massage session. Additionally, future research should concentrate on exploring chronic massage interventions across various muscles in the back, neck, upper, and lower limbs. It is recommended that these studies incorporate a range of massage types, durations, intensities, and weekly frequencies to comprehensively understand the efficacy and optimize the parameters of massage therapy for alleviating muscle stiffness and potentially addressing chronic pain conditions.

## Data Availability

The raw data supporting the conclusions of this article will be made available by the authors, without undue reservation.
