# Supplementary material for: The effect of a 5-week therapeutic massage on erector spinae and upper trapezius muscle stiffness as determined by shear-wave elastography: a randomized controlled trial
Source: Front Sports Act Living. 2024 Aug 26;6:1428301. doi: 10.3389/fspor.2024.1428301 (PMC11381258; doi:10.3389/fspor.2024.1428301)
Supplement: Supplementary file 1 [file Table1.docx]

**Supplementary file 1.** Reliability of the measurements across muscles, locations, sides and study timeframe.

| **Muscle/Location/Side** | **Timeframe** | Descriptive statistics | | | | Reliability | | | |
| --- | --- | --- | --- | --- | --- | --- | --- | --- | --- |
|  |  | Mean 1 | SD | Mean 2 | SD | ICC | 95% CI | | |
| ES, inferior, right | Baseline | 16.97 | 2.78 | 16.77 | 2.62 | 0.89 | | 0.79 | 0.95 |
| ES, inferior, left | Baseline | 15.49 | 2.71 | 15.45 | 2.94 | 0.83 | | 0.67 | 0.91 |
| ES, superior, right | Baseline | 15.41 | 3.63 | 15.44 | 3.89 | 0.93 | | 0.86 | 0.97 |
| ES, superior, left | Baseline | 15.86 | 4.31 | 15.83 | 3.96 | 0.94 | | 0.88 | 0.97 |
| UT, distal, right | Baseline | 7.58 | 1.91 | 7.50 | 1.90 | 0.87 | | 0.74 | 0.94 |
| UT, distal, left | Baseline | 7.64 | 1.73 | 7.27 | 1.87 | 0.83 | | 0.68 | 0.92 |
| UT, proximal, right | Baseline | 7.13 | 2.01 | 7.08 | 2.16 | 0.90 | | 0.80 | 0.95 |
| UT, proximal, left | Baseline | 7.33 | 2.08 | 7.17 | 1.94 | 0.77 | | 0.57 | 0.88 |
| ES, inferior, right | Post-intervention | 16.15 | 3.12 | 16.14 | 3.18 | 0.93 | | 0.86 | 0.97 |
| ES, inferior, left | Post-intervention | 14.87 | 2.74 | 15.03 | 2.32 | 0.89 | | 0.79 | 0.95 |
| ES, superior, right | Post-intervention | 14.62 | 3.74 | 14.61 | 3.85 | 0.98 | | 0.96 | 0.99 |
| ES, superior, left | Post-intervention | 14.73 | 3.80 | 14.88 | 4.20 | 0.98 | | 0.95 | 0.99 |
| UT, distal, right | Post-intervention | 7.42 | 1.72 | 7.22 | 1.55 | 0.89 | | 0.79 | 0.95 |
| UT, distal, left | Post-intervention | 7.97 | 2.14 | 7.83 | 2.01 | 0.95 | | 0.91 | 0.98 |
| UT, proximal, right | Post-intervention | 6.58 | 2.29 | 6.57 | 2.30 | 0.95 | | 0.90 | 0.98 |
| UT, proximal, left | Post-intervention | 6.46 | 2.34 | 6.66 | 2.38 | 0.95 | | 0.89 | 0.97 |
| ES, inferior, right | Follow-up | 15.81 | 2.64 | 15.89 | 3.25 | 0.92 | | 0.84 | 0.96 |
| ES, inferior, left | Follow-up | 14.79 | 2.54 | 14.88 | 2.70 | 0.94 | | 0.87 | 0.97 |
| ES, superior, right | Follow-up | 15.22 | 3.71 | 15.47 | 4.25 | 0.94 | | 0.88 | 0.97 |
| ES, superior, left | Follow-up | 15.02 | 4.78 | 14.59 | 4.38 | 0.99 | | 0.97 | 0.99 |
| UT, distal, right | Follow-up | 7.51 | 1.79 | 7.47 | 1.88 | 0.93 | | 0.87 | 0.97 |
| UT, distal, left | Follow-up | 7.66 | 1.76 | 7.70 | 1.73 | 0.90 | | 0.81 | 0.95 |
| UT, proximal, right | Follow-up | 5.87 | 1.46 | 5.98 | 1.69 | 0.88 | | 0.76 | 0.94 |
| UT, proximal, left | Follow-up | 6.13 | 1.36 | 6.29 | 1.25 | 0.81 | | 0.63 | 0.90 |
